# METTL14-mediated N6-methyladenosine modification induces the ferroptosis of hypoxia/reoxygenation-induced cardiomyocytes

**DOI:** 10.1186/s13019-024-02711-0

**Published:** 2024-04-25

**Authors:** Chunyu Zhao, Jianing Li

**Affiliations:** Department of General Practice, The Fourth Affiliated Hospital of Harbin Medical University, No.37, Yiyuan Street, Nangang District, Harbin, Heilongjiang 150000 China

**Keywords:** Hypoxia reoxygenation, METTL14, Ferroptosis, miR-146a-5p, APPL1, Cardiomyocytes

## Abstract

**Background:**

Hypoxia/reoxygenation (H/R) induces cardiomyocyte ferroptosis, a core remodeling event in myocardial ischemia/reperfusion injury. Methyltransferase-like 14 (METTL14) emerges as a writer of N6-methyladenosine (m6A) modification. This study was conducted to decipher the role of METTL14 in H/R-induced cardiomyocyte ferroptosis.

**Methods:**

Mouse cardiomyocytes HL-1 were cultured and underwent H/R treatment. The degree of ferroptosis after H/R treatment was appraised by the cell counting kit-8 assay, assay kits (ROS/GSH/Fe^2+^), and Western blotting (GPX4/ACSL4). The intracellular expressions of METTL14, pri-miR-146a-5p, miR-146a-5p, or adaptor protein phosphotyrosine interacting with PH domain and leucine zipper 1 (APPL1) were examined by real-time quantitative polymerase chain reaction or Western blotting, with m6A quantification analysis and RNA immunoprecipitation to determine the total m6A level and the expression of pri-miR-146a-5p bound to DiGeorge critical region 8 (DGCR8) and m^6^A-modified pri-miR-146a-5p. The binding of miR-146a-5p to APPL1 was testified by the dual-luciferase assay.

**Results:**

H/R treatment induced cardiomyocyte ferroptosis (increased ROS, Fe^2+^, and ACSL4 and decreased GSH and GPX4) and upregulated METTL14 expression. METTL14 knockdown attenuated H/R-induced cardiomyocyte ferroptosis. METTL14 induced the recognition of pri-miR-146a-5p by DGCR8 by increasing m^6^A modification on pri-miR-146a-5p, which promoted the conversion of pri-miR-146a-5p into miR-146a-5p and further repressed APPL1 transcription. miR-146a-5p upregulation or APPL1 downregulation limited the inhibitory effect of METTL14 downregulation on H/R-induced cardiomyocyte ferroptosis.

**Conclusion:**

METTL14 promoted miR-146a-5p expression through the recognition and processing of pri-miR-146a-5p by DGCR8, which repressed APPL1 transcription and triggered H/R-induced cardiomyocyte ferroptosis.

## Background

Ischemic heart disease represents a leading cause of medical burden worldwide [1–3]. Myocardial ischemia occurs when the coronary artery is obstructed with reduced blood supply to the dismal ischemic region causing heart dysfunction. Reperfusion is an essential remedy after ischemia but will further aggravate ischemic injury, which is termed as myocardial ischemia/reperfusion (I/R) injury [4, 5]. The pathological background of myocardial I/R injury is associated with multiple cellular events, such as apoptosis, inflammation, oxidative stress, and mitochondrial dysfunction [5–7]. In particular, ferroptosis, a novel programmed cell death characterized by ferric ion (Fe^2+^) overload and lipid peroxidation, participates in numerous diseases, including myocardial I/R injury [8, 9]. Ferroptosis-associated reactive oxygen species (ROS) accumulation and Fe^2+^ overload are important causes of cardiomyocyte injury and the application of ferroptosis inhibitors can effectively alleviate cardiomyocyte death induced by I/R injury [10]. For this reason, it remains necessary to explore the molecular route of ferroptosis to provide novel insights for the treatment of myocardial I/R injury.

As one of the most popular modifications on messenger RNA (mRNA), N6-methyladenosine (m6A) is prevalent in the transcriptome of most RNAs and dynamically regulates a variety of cellular processes, and human diseases like reproductive system diseases, acute lung injury, aortic dissection, and viral infection [11–15]. Such modification is encoded by “writers” [methyltransferases, methyltransferase-like 14 (METTL14), METTL3, and WT1 associated protein (WTAP)], “erasers” (demethylases, FTO and alkB homolog 5), and “readers” (YTH N6-methyladenosine RNA binding protein 1/2/3, YTH domain containing 1/2) [16]. An increase in m6A modification has been observed in cardiomyocytes and neurons after hypoxia/reoxygenation (H/R) treatment [17, 18]. The in vitro H/R model is a commonly used model to simulate I/R injury in vivo and is widely used in the study of many diseases, such as I/R-induced acute kidney injury, myocardial I/R injury, and intestinal I/R injury [19–21]. What’s more, as a m6A writer, the mRNA levels of METTL14 have been reported to be augmented in H/R-treated cardiomyocytes [18]. However, the role of METTL14 in H/R-induced cardiomyocyte ferroptosis remains ambiguous.

DiGeorge syndrome critical region 8 (DGCR8) is a microprocessor protein that recognizes and processes primary (pri)-microRNAs (miRNAs). METTL3 or METTL14 catalyzes m6A modification on pri-miRNAs to facilitate the recognition of pri-miRNAs by DGCR8, upon which DGCR8 transforms pri-miRNA into precursor miRNAs, resulting in increased expression of mature miRNA [22]. Altered miRNA expression is verified to regulate the progression of I/R injury [23]. One miRNA signature, namely miR-146a-5p, has been documented as a regulator of cardiac damage and cancer [24–27]. Besides, METTL14/METTL3 can regulate miR-146a-5p in a m6A-dependent manner [17, 18], suggesting a potential correlation between METTL14 and miR-146a-5p. However, there are conflicting shreds of evidence about its role in myocardial I/R injury, with one reporting its protective role and the other illustrating its detrimental role [28, 29]. Therefore, the role of miR-146a-5p in myocardial I/R injury warrants further validation. Adaptor protein phosphotyrosine interacting with PH domain and leucine zipper 1 (APPL1) is a vital modulator of insulin and adiponectin signaling [30]. By interacting with AMP-activated protein kinase signaling or binding to adiponectin receptor, APPL1 can attenuate H/R-induced apoptosis, inflammation, and ROS generation in myocardial I/R injury [31, 32]. miRNAs can induce mRNA decay through recognition of the 3’ untranslated region and APPL1 was identified as a target gene of miR-146a-5p using multiple databases, suggesting the regulatory role of miR-146a-5p in APPL1 biogenesis in myocardial I/R injury.

In the same light, the objective of our study was to unravel the regulatory mechanism of METTL14 in H/R-induced cardiomyocyte ferroptosis through interaction with the miR-146a-5p/APPL1 axis, hoping to provide a novel rationale for the treatment of myocardial I/R injury.

## Methods

### Cell culture

Mouse cardiomyocytes HL-1 were procured from ATCC (Manassas, VA, USA) and were cultured in Dulbecco’s modified Eagle medium (Sigma, St. Louis, MO, USA) containing 10% fetal bovine serum, penicillin (100 U/mL)/streptomycin (100 µg/mL) After that, cells were preserved in an incubator under a condition of 37℃, 5% CO_2_, and 95% air.

### Cell treatment

Small interfering RNAs of METTL14 (si-METTL14), APPL1 (si-APPL1), and their negative control (si-NC) were synthesized and obtained from GenePharma (Shanghai, China). miR-146a-5p mimics and mimics NC were also provided by GenePharma. As prescribed by the manufacturer’s protocol, Lipofectamine 3000 (Invitrogen, Carlsbad, CA, USA) was employed to transfect the above vectors. In brief, HL-1 cells were cultured until 60–80% confluence and transfected with vectors. After transfection, HL-1 cells were subjected to H/R treatment. During H/R treatment, 1 × 10^5^ cells were seeded into the 6-well plates and were cultured in an anoxic chamber (94% N_2_, 5% CO_2_, and 1% O_2_) for 16 h, followed by 6 h culture under a normal oxygen environment of 5% CO_2_ for reoxygenation [33, 34]. Cells in the control group were cultured under conventional conditions.

### Cell counting kit-8 (CCK-8) assay

HL-1 cells were loaded into the 96-well plates (1 × 10^4^/mL). Each well was incorporated with 100 µL cell suspension. After treatment, each well was incorporated with 10 µL of CCK-8 solution (Beyotime, Haimen, China) and was cultured for 3 h at 37℃. The absorbance at a wavelength of 450 nm was measured with the assistance of a microplate reader (Thermo Fisher Scientific, Waltham, MA, USA).

### Glutathione (GSH) and ROS measurements

The intracellular levels of GSH were determined using a standard GSH assay kit (Nanjing Jiancheng Bioengineering Institute, Nanjing, China) in accordance with the producer’s protocol. HL-1 cells were loaded into the 24-well plates (1 × 10^5^ cells/well) and cultured overnight. Treated HL-1 cells were collected and broken. The supernatant was fully mixed with precipitator, buffer, and developer and was stayed for 5 min, with a microplate reader to determine absorbance (at 405 nm). The ultimate results were presented as normalized results.

The intracellular levels of ROS were determined by applying a standard ROS assay kit (ab113851, Abcam, Cambridge, MA, USA) following the producer’s protocol. HL-1 cells were seeded into the 96-well plates at a density of 2.5 × 10^4^ cells/well. Treated or transfected cells were cultured with ROS working solution at 37 °C in the dark for 45 min. The fluorescence intensity was evaluated, with ultimate results shown as percentages (% of Control).

### Fe^2+^ content measurement

For measurement of Fe^2+^ content, HL-1 cells were harvested and homogenized with Phosphate Buffer Saline and centrifuged at 12,000 g and 4℃ for 10 min. According to the producer’s protocol, Fe^2+^ concentration in the supernatant was determined using a standard Fe^2+^ content assay kit (ab83366, Abcam). Simply put, standards (50 µL) or samples (50 µL) were blended with quantichrom working reagent (200 µL) in a 96-well plate and were incubated at ambient temperature overnight. The Fe^2+^ levels were calculated with a microplate reader to determine optical density (at 590 nm).

### m6A quantification analysis

The RNA in cells was separated using the TRIzol reagent (Invitrogen) by strictly following the producer’s protocol. The m6A content in RNA was analyzed with application of a EpiQuikTM m6A RNA methylation quantification assay kit (colorimetric method, Farmingdale, NY, USA). In brief, test wells were added with 100–300 ng RNA, with capture and determination antibody solution added into each well respectively. The m6A content was determined according to sample absorbance at 450 nm.

### RNA immunoprecipitation (RIP)

The total RNA was separated from HL-1 cells through TRIzol treatment. Subsequently, the antibody against m6A (ab208577, Abcam) or DGCR8 (ab191875, Abcam) or immunoglobulin G (ab172730, Abcam) was coupled with protein A/G magnetic beads in IP buffer (140 nM NaCl, 1% NP-40, 2 mm ethylene diamine tetraacetic acid, 20 mm Tris pH 7.5) overnight at 4℃. Immunoprecipitated RNA was eluted from microbeads and reverse-transcribed for RT-qPCR.

### Bioinformatics

The downstream mRNAs of miR-146a-5p were analyzed with the help of Starbase (https://starbase.sysu.edu.cn/index.php) [35], Targetscan (https://www.targetscan.org/vert_71/) [36], miRDB (http://mirdb.org/index.html) [37], and miRWalk databases (http://mirwalk.umm.uni-heidelberg.de/) [38]. The binding site of miR-146a-5p and APPL1 was attained from the Starbase database.

### Dual-luciferase assay

HL-1 cells were planted into the 96-well plates and were cultured until 60% confluence before transfection. Wildtype (WT) and mutant-type (MUT) APPL1 fragments containing the binding site of miR-146a-5p were inserted into pGL3 reporter gene vectors (Promega Corporation, Madison, WI, USA). The constructed vectors were co-transfected miR-146a-5p mimics or mimics NC into HL-1 cells. After 48 h, the luciferase activity was quantified by employing a luciferase assay kit (Promega).

### Real-time quantitative polymerase chain reaction (RT-qPCR)

After H/R induction, HL-1 cells were collected for RT-qPCR. The total RNA was obtained by TRIzol treatment and its purity was determined with a NanoDrop ND-1000 spectrophotometer (Thermo Fisher Scientific) to measure the absorption ratio at 280 nm and 260 nm. The reverse transcription of RNA into the complementary DNA and qPCR was conducted with application of a PrimeScript™ RT reagent kit with genomic DNA eraser (RR047A, Takara, Tokyo, Japan) and a SYBR™ Green quantitative kit (Thermo Fisher Scientific). With U6 serving as the internal reference of miR-146a-5p [28] and glyceraldehyde-3-phosphate dehydrogenase 9 (GAPDH) serving as the internal reference of mRNA, the relative gene expression was quantified based on the 2^-ΔΔCt^ method [39]. Information on primers is shown in Table 1.

**Table 1:** PCR primer sequences

Gene	Sequence (5’-3’)
METTL14	F: GCACAGACGGGGACTTCATT
	R: TCCCAAAGAGATGAAGGCGT
miR-146a-5p	F: GCGGTCGTGAGAACTGAATTC
	R: GTGTCGTGGAGTCGGCAATT
pri-miR-146a-5p	F: CAGGTATCACTGGGGAACGG
	R: TCTTCACGTCAGCAAGAGCA
APPL1	F: GAAAAACAGCGTTTTCCTTTG
	R: TGCATGACAAGAACTAAGCTC
U6	F: GCTCGCTTCGGCAGCACATATA
	R: GGAACGCTTCACGAATTTGCG
GAPDH	F: GGTCCCAGCTTAGGTTCATCA
	R: AATCCGTTCACACCGACCTT

### Western blot assay

HL-1 cells were incubated with the radioimmunoprecipitation assay buffer (Beijing Solarbio Science & Technology Co., Ltd.) containing 1% phenylmethanesulfonylfluoride on ice for 30 min and were centrifuged at 4℃ and 12,000 g for 15 min to harvest the supernatant, with the bicinchoninic acid method (Beyotime) to determine protein concentration. Protein was separated using 12% sodium dodecyl sulfate polyacrylamide gel electrophoresis and transferred onto polyvinylidene fluoride membranes (Millipore, Billerica, MA, USA). Following 2 h blockade with 5% nonfat milk powder solution, the membrane incubation was conducted with primary antibodies against GPX4 (1:500, PA5-102521), ACSL4 (1:500, PA5-27137), METTL14 (1:500, PA5-117138), APPL1 (1:2000, MA5-26917), and GAPDH at 4℃, followed by three rinses with Tris Buffered Saline Tween, with secondary antibody (1:300, 65-6120) for 2 h incubation at ambient temperature. Following these steps, protein was visualized using a chemiluminescence assay kit (Advansta, Inc., Menlo Park, CA, USA), with Quantity One software (v4.6.6, Bio-Rad, Hercules, CA, USA) to analyze the grayscale of protein bands. The grayscale of target proteins was normalized to GAPDH and was presented as the relative expression of target proteins. All antibodies were provided by Thermo Fisher Scientific.

### Statistical analysis

All data were handled by SPSS21.0 statistical software (IBM SPSS Statistics, Chicago, IL, USA) and GraphPad Prism 8.0 software (GraphPad Software Inc., San Diego, CA, USA) aiming for statistical analysis and graphing. Data were in accordance with normal distribution and equal variance. Data in two panels were analyzed by the *t* test and data in multiple panels were analyzed by one-way or two-way analysis of variance (ANOVA), with Tukey’s multiple comparison test used for post hoc test. *P* < 0.05 was indicative of differences with statistical significance.

## Results

### METTL14 downregulation inhibits H/R-induced ferroptosis

**Fig. 1:**
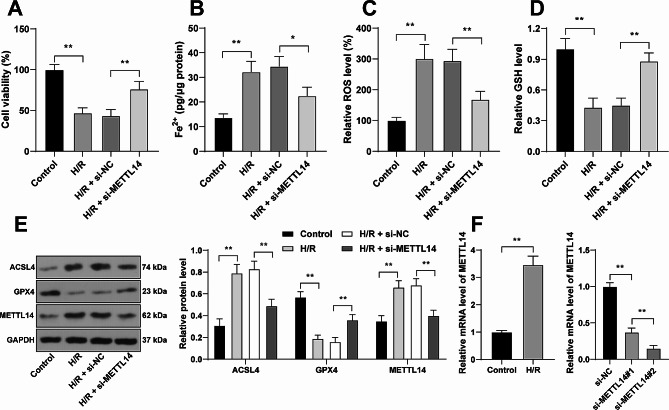
METTL14 downregulation inhibits H/R-induced ferroptosis. HL-1 cells were transfected with si-METTL14, with si-NC as negative control, followed by H/R induction. **A**: Cell viability was evaluated by the CCK-8 assay; **B**-**D**: Fe^2+^ content (**B**), ROS levels (**C**), and GSH levels (**D**) were determined by assay kits; **E**: Protein levels of ACSL4, GPX4, and METTL14 were determined by Western blot assay; **F**: METTL14 mRNA levels were determined by RT-qPCR. Each experiment was repeated three times independently. Data were shown as mean ± standard deviation. Data in panel F (left) were analyzed by the *t* test, data in panels A-D and F (right) were analyzed by one-way ANOVA, data in panel E were analyzed by two-way ANOVA, followed by Tukey’s multiple comparison test. * *P* < 0.05, ** *P* < 0.01

To explore the role of METTL14 in H/R-induced ferroptosis of cardiomyocytes, HL-1 cells were treated with H/R induction. After H/R, cell viability was decreased (*P* < 0.01, Fig. 1A), Fe^2+^ content was increased (*P* < 0.01, Fig. 1B), ROS levels and ACSL4 protein levels were elevated, while GSH levels and GPX4 protein levels were reduced (*P* < 0.01, Fig. 1C-E), suggesting that H/R induced the ferroptosis of cardiomyocytes. The expression levels of METTL14 were determined and were found to be upregulated by H/R induction (*P* < 0.01, Fig. 1E-F). Next, the transfection with si-METTL14 successfully resulted in the downregulation of METTL14, and si-METTL14#2 with higher silencing efficiency was used for the subsequent experiments (*P* < 0.01, Fig. 1E-F). Upon METTL14 downregulation, cell viability was amplified and Fe^2+^ content was reduced (*P* < 0.05, Fig. 1A-B), ROS levels and ACSL4 protein levels were decreased, while GSH levels and GPX4 protein levels were elevated (*P* < 0.01, Fig. 1C-E). These findings elicited that METTL14 downregulation inhibited H/R-induced ferroptosis.

### METTL14-mediated m6A modification promotes the transition of pri-miR-146a-5p into miR-146a-5p

**Fig. 2:**
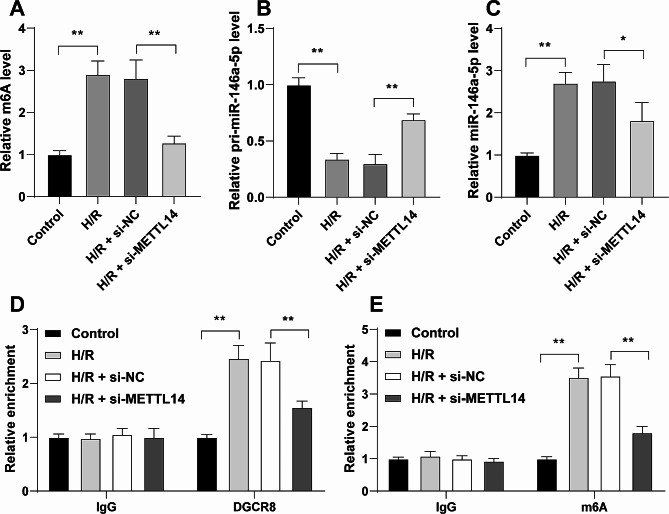
METTL14-mediated m6A modification promotes the transition of pri-miR-146a-5p into miR-146a-5p. **A**: m6A levels in cells were determined by a m6A quantification assay kit; **B**-**C**: pri-miR-146a-5p and miR-146a-5p expression levels in cells were determined by RT-qPCR; **D**-**E**: The binding of DGCR8 and m6A to pri-miR-146a-5p was analyzed by the RIP assay. Each experiment was repeated three times independently. Data were shown as mean ± standard deviation. Data in panels A-C were analyzed by one-way ANOVA and data in panels D-E were analyzed by two-way ANOVA, followed by Tukey’s multiple comparison test. * *P* < 0.05, ** *P* < 0.01

As indicated by previous studies, METTL14-mediated m6A modification regulates the processing and maturation of pri-miRNAs by DGCR8 to function in disease progression [40], METTL14 can regulate miR-146a-5p expression [41], and miR-146a-5p is expressed at high levels after H/R treatment [28]. m6A levels were determined and were found to be increased after H/R treatment and decreased in response to METTL14 downregulation (*P* < 0.01, Fig. 2A). In addition, H/R treatment downregulated the expression levels of pri-miR-146a-5p and upregulated the expression levels of miR-146a-5p, while METTL14 downregulation resulted in the opposite trends (*P* < 0.05, Fig. 2B-C). DGCR8-bound pri-miR-146a-5p levels and m6A-modified pri-miR-146a-5p levels were observed to be reduced in cells of the si-METTL14 group (*P* < 0.01, Fig. 2D-E). Collectively, METTL14-mediated m6A modification promoted DGCR8-mediated processing and maturation of pri-miR-146a-5p and upregulated miR-146a-5p expression in H/R-induced cardiomyocytes.

### miR-146a-5p overexpression neutralizes the inhibitory role of METTL14 downregulation in the ferroptosis of H/R-induced cardiomyocytes

**Fig. 3:**
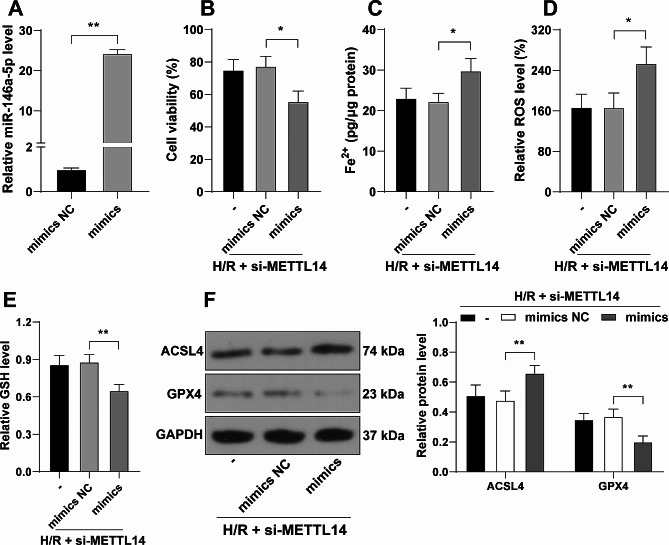
miR-146a-5p overexpression neutralizes the inhibitory role of METTL14 downregulation in the ferroptosis of H/R-induced cardiomyocytes. HL-1 cells were transfected with miR-146a-5p mimics, with mimics NC as the negative control. **A**: miR-146a-5p expression levels were determined by RT-qPCR; **B**: Cell viability was evaluated by the CCK-8 assay; **C**-**E**: Fe^2+^ content (**C**), ROS levels (**D**), and GSH levels (**E**) were determined by assay kits; F: Protein levels of ACSL4 and GPX4 in cells were determined by Western blot assay. Each experiment was repeated three times independently. Data were shown as mean ± standard deviation. Data in panel A were analyzed by the *t* test, data in panels **B**-**E** were analyzed by one-way ANOVA, data in panel F were analyzed by two-way ANOVA, followed by Tukey’s multiple comparison test. * *P* < 0.05, ** *P* < 0.01

Next, miR-146a-5p expression levels were successfully elevated (*P* < 0.01, Fig. 3A), accompanied by combined treatment with si-METTL14. After H/R induction, miR-146a-5p overexpression notably reduced cell viability (*P* < 0.05, Fig. 3B). Compared to METTL14 downregulation alone, the combined treatment effectively promoted the ferroptosis of H/R-induced cardiomyocytes (*P* < 0.05, Fig. 3C-F).

### miR-146a-5p targets and inhibits APPL1 transcription

**Fig. 4:**
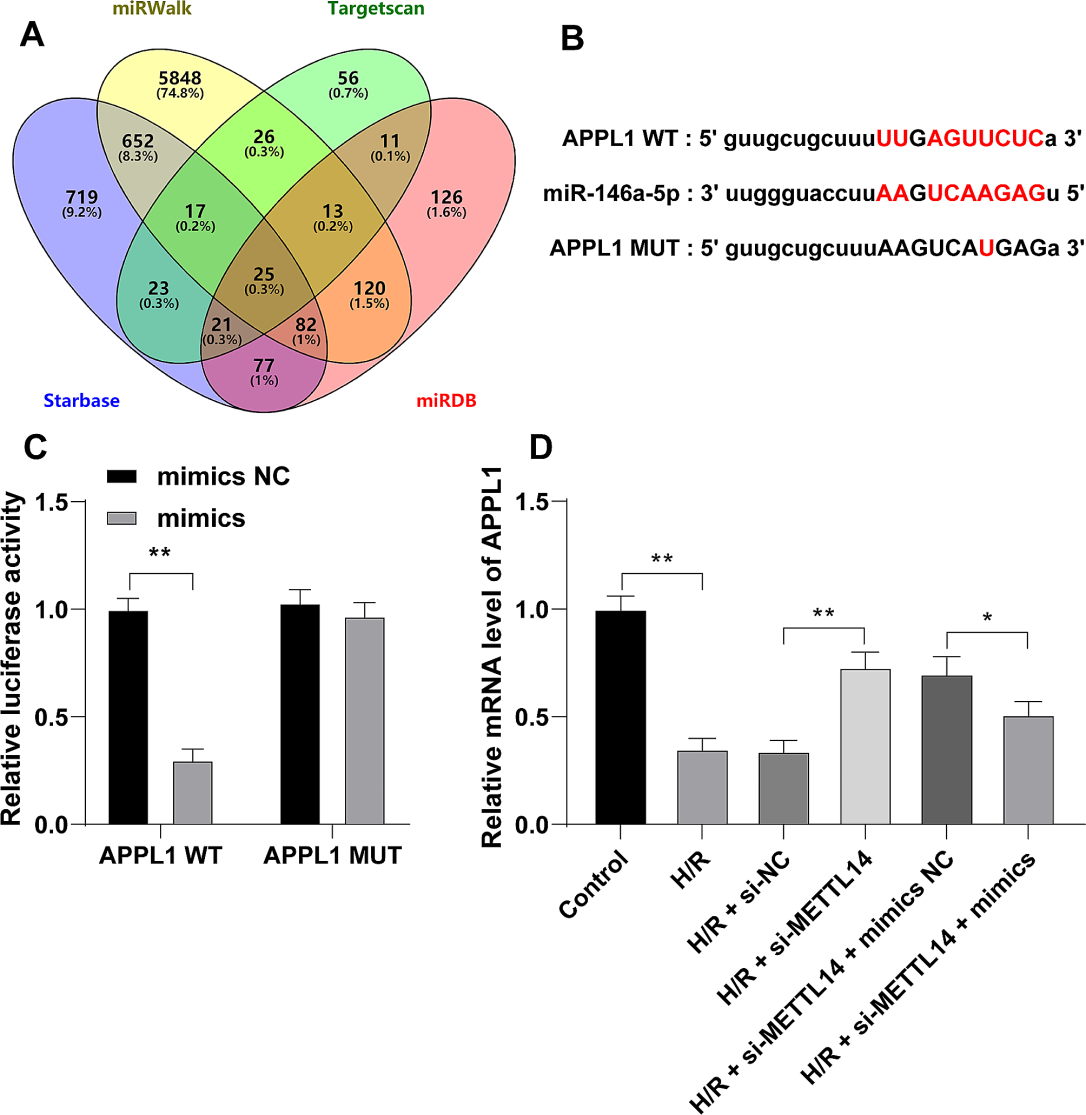
miR-146a-5p targets and inhibits APPL1 transcription. **A**: The downstream target genes of miR-146a-5p were predicted on the databases and intersections were identified; **B**: The binding site of miR-146a-5p and APPL1; **C**: The binding of miR-146a-5p to APPL1 was analyzed by the dual-luciferase assay; D: mRNA levels of APPL1 in cells were determined by RT-qPCR. Each experiment was repeated three times independently. Data were shown as mean ± standard deviation. Data in panel D were analyzed by one-way ANOVA and data in panel C were analyzed by two-way ANOVA, followed by Tukey’s multiple comparison test. * *P* < 0.05, ** *P* < 0.01

The downstream target genes of miR-146a-5p were predicted on the databases Starbase, Targetscan, miRDB, and miRWalk, and the intersections of predicted targets were identified (Fig. 4A). Among intersections, the downregulation of APPL1 after H/R treatment has been reported previously [31, 32]. Therefore, the dual-luciferase assay was conducted according to the binding site (Fig. 4B) and the co-transfection of miR-146a-5p mimics and APPL1 WT markedly reduced the luciferase activity, indicating the targeted binding between miR-146a-5p and APPL1 (*P* < 0.01, Fig. 4C). The mRNA levels of APPL1 were determined and were found to be reduced by both H/R and miR-146a-5p mimics treatments and elevated by si-METTL14 treatment (*P* < 0.05, Fig. 4D). Altogether, our findings suggested that METTL14-mediated m6A modification promoted miR-146a-5p expression and further inhibited APPL1 transcription.

### APPL1 downregulation neutralizes the inhibitory role of METTL14 downregulation in the ferroptosis of H/R-induced cardiomyocytes

**Fig. 5:**
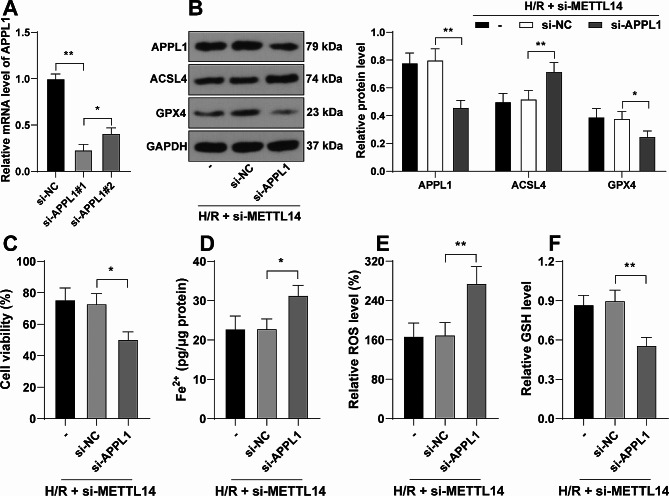
APPL1 downregulation neutralizes the inhibitory role of METTL14 downregulation in the ferroptosis of H/R-induced cardiomyocytes. HL-1 cells were transfected with si-APPL1, with si-NC as the negative control. **A**: mRNA levels of APPL1 in cells were determined by RT-qPCR; **B**: Protein levels of APPL1, ACSL4, and GPX4 in cells were determined by Western blot assay; **C**: Cell viability was evaluated by the CCK-8 assay; **D**-**F**: Fe^2+^ content (**D**), ROS levels (**E**), and GSH levels (**F**) were determined by assay kits. Each experiment was repeated three times independently. Data were shown as mean ± standard deviation. Data in panels A and C-F were analyzed by one-way ANOVA, and data in panel B were analyzed by two-way ANOVA, followed by Tukey’s multiple comparison test. * *P* < 0.05, ** *P* < 0.01

Eventually, APPL1 expression was knocked down in HL-1 cells, and si-APPL1#1 with high knockdown efficiency was combined with si-METTL14 for the rescue experiments (*P* < 0.05, Fig. 5A-B). Our results showed that APLL1 downregulation notably inhibited the viability of H/R-induced cardiomyocytes and promoted ferroptosis (*P* < 0.05, Fig. 5B-F).

## Discussion

**Fig. 6:**
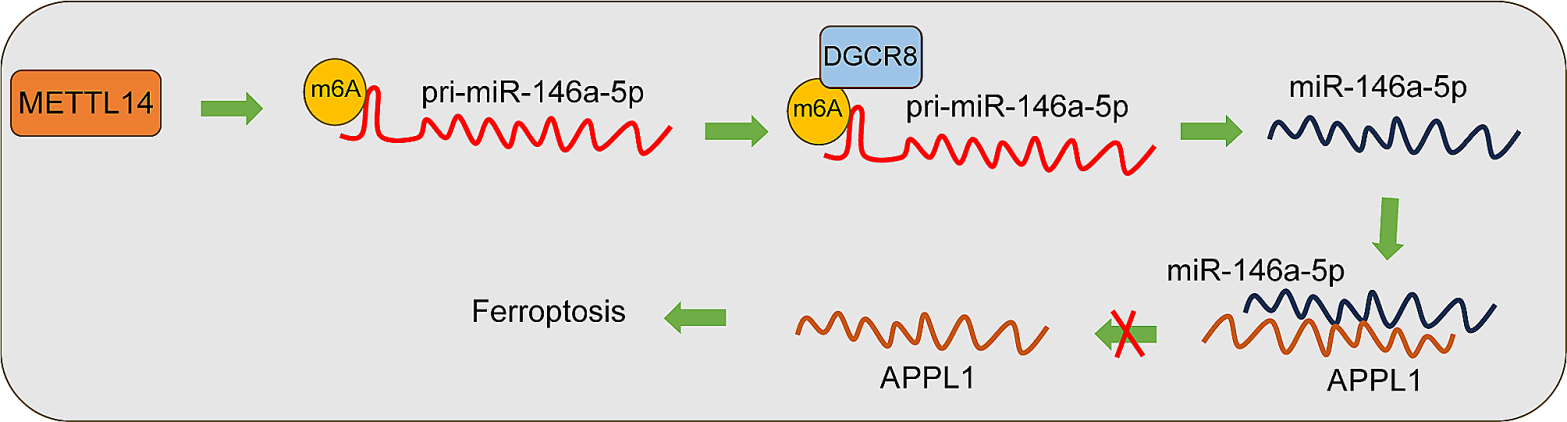
Role and mechanism of METTL14 in regulating H/R-induced ferroptosis in cardiomyocytes. METTL14 induces the recognition and processing of pri-miR-146a-5p by DGCR8 by up-regulating the m6A methylation level on pri-miR-146a-5p, which promotes the expression of miR-146a-5p, which in turn targets and represses the transcriptional level of APPL1 and promotes H/R-induced ferroptosis in cardiomyocytes

Myocardial I/R injury is considered an inescapable risk event accompanied by myocardial ischemia and results in poor cardiac outcomes through multiple cellular and molecular events [42]. Ferroptosis is a core remodeling event after myocardial I/R injury and brings the new gospel to the prevention and management of the disease [8]. Post-translational modifications, such as histone methylation, acetylation, phosphorylation, ubiquitylation, SUMOylation, noncoding RNAs, and m6A modification, participate in the regulation of myocardial I/R injury [43, 44]. In the current study, we identified METTL14, miR-146a-5p, and APPL1 as regulators of H/R-induced cardiomyocyte ferroptosis and unified them into a novel molecular route wherein METTL14 upregulates m6A methylation on pri-miR-146a-5p to induce the recognition and processing of pri-miR-146a-5p by DGCR8 leading to increased miR-146a-5p expression and further inhibits APPL1 transcription, consequently promoting H/R-induced cardiomyocyte ferroptosis (Fig. 6).

Ferroptosis has drawn increasing attention due to its great potential for the treatment of complex diseases. Its occurrence involves Fe^2+^ overload, intracellular GSH ablation, and reduced GPX4 activity, which leads to ROS generation and lipid peroxidation [45]. ACSL4, an isoform of the Acyl-CoA synthetase long-chain family, acts as a biomarker of ferroptosis due to its contribution to lipid intermediate accumulation [46]. In our study, H/R treatment was adopted to mimic myocardial I/R injury in vitro and induced ferroptosis as evidenced by increased intracellular Fe^2+^ content, ROS, and ACSL4 levels and reduced GSH and GPX4 levels. Ferroptosis entails m6A modification aiming for the regulation of non-coding RNA biogenesis [47]. Additionally, there is a growing body of evidence that WTAP and METTL3-mediated m6A modification is activated and further aggravates myocardial I/R injury, while curcuminoids reduce the total m6A modification as a response to the disease treatment [18, 48, 49], suggesting that m6A modification is a negative regulator of myocardial I/R injury. As one of m6A writer, METTL14 knockdown has been known to protect the heart from fibrosis and I/R injury [50]. Moreover, METTL14 is likely to exacerbate doxorubicin-induced cardiomyocyte ferroptosis [51]. In accordance, H/R treatment robustly upregulated METTL14, and the knockdown by si-METTL14#2 increased cell viability and attenuated ferroptosis. Our results and existing pieces of evidence substantiated that METTL14 inhibitor may be a remedy to alleviate myocardial I/R injury by quenching ferroptosis.

Different types of noncoding RNAs, including miRNAs, long noncoding RNAs, and circular RNAs, play critical roles in myocardial injury through interaction with their molecular targets [52]. In particular, miRNAs are a major source of targets for myocardial injury. m6A writers can cooperate with DGCR8 to promote DGCR8-dependent recognition and processing of pri-miRNAs, increasing the expression of mature miRNAs [22]. METTL3 and METTL14 have been shown to regulate miR-146a-5p expression in a m6A-dependent manner [41, 53]. The mechanism of miR-146a-5p in myocardial pathologies is rather complex. One hypothesis suggests that miR-146a-5p promotes cardiomyocyte apoptosis, inflammation, and myocardial infarction (MI)-induced cardiac dysfunction [25, 54, 55]. Another hypothesis maintains that miR-146a-5p exerts cardioprotective by reducing fibrosis and promoting cardiac repair [56, 57]. Prominently, the role of miR-146a-5p in myocardial I/R injury is controversial. Troxerutin-induced repression of miR-146a-5p moderates cardiomyocyte apoptosis in myocardial I/R injury [28], whereas miR-146a-5p upregulation by silencing of long noncoding RNA SRY-box transcription factor 2 overlapping transcript also contributes to the remission of this disease [29]. Intriguingly, miR-146a-5p can mediate a compensatory regulation that protects cardiac function after MI, but this compensatory regulation is supposed to be inhibited by aging, leading to elevated susceptibility to myocardial I/R injury [58]. Based on our results, the total m6A was increased after H/R treatment, which may be due to METTL14-mediated m6A modification on pri-miR-146a-5p. In addition, our results revealed that elevated m6A promoted the processing and maturation of miR-146a-5p by DGCR8, resulting in increased miR-146a-5p expression. Subsequently, miR-146a-5p gain-of-expression reversed the inhibition of H/R-induced cardiomyocyte ferroptosis caused by METTL14 downregulation.

APPL1 plays a role in various organs, including vasculature, heart, lung, and colon, because of its regulation of the adiponectin signaling [59]. Interestingly, the expression of APPL1 can be altered by miRNAs, such as miR-340-5p [60]. APPL1 is known to protect against cardiomyocyte senescence, cardiac fibrosis, and diabetic cardiomyopathy [61–63]. More importantly, APPL1 can function as a potential therapeutic target for myocardial I/R injury by antagonizing apoptosis, oxidative stress, cytotoxicity, and release of inflammatory cytokines [31, 32, 64]. In the same light, our experimentation uncovered that the downregulation of APPL1 mRNA was found in H/R-induced cardiomyocytes and miR-146a-5p mimics potently repressed APPL1 transcription. APPL1 loss-of-expression significantly abrogated the inhibitory role of METTL14 downregulation in H/R-induced cardiomyocyte ferroptosis.

## Conclusions

To summarize, our study is the pioneer to unravel METTL14-mediated negative regulation of H/R-induced cardiomyocyte ferroptosis, which is associated with DGCR8-depedent upregulation of miR-146a-5p and inhibition of APPL1 transcription. Our findings confer a theoretical foundation for the clinical study of these molecules in myocardial I/R injury, which may develop a new strategy for clinical management. However, the validation of our mechanism is limited at present due to (1) a lack of animal experiments, (2) only exploration of a single downstream pathway for METTL14 in H/R-induced cardiomyocytes, and (3) no interpretation of other downstream targets of miR-146a-5p, except APPL1. With future endeavors, other downstream mechanisms of METTL14 are warranted to be explored and our mechanism is warranted to be validated in vivo, while the reasons for the high expression of METTL14 and the downstream target genes of miR-146a-5p will be validated to fully reveal the role of the METTL14/miR-146a-5p axis in I/R-induced myocardial injury, so as to provide more theoretical references for the treatment of myocardial I/R injury.

## Data Availability

The data that support this study are available from the corresponding author upon reasonable request.

## Abbreviations

I/R: ischemia/reperfusion
Fe^2+^: ferric ion
ROS: reactive oxygen species
m6A: N6-methyladenosine
mRNA: messenger RNA
METTL14: methyltransferase-like 14
WTAP: WT1 associated protein
H/R: hypoxia/reoxygenation
DGCR8: DiGeorge syndrome critical region 8
miRNAs: microRNAs
pri-miRNAs: primary microRNAs
APPL1: Adaptor protein phosphotyrosine interacting with PH domain and leucine zipper 1
RT-qPCR: real-time quantitative polymerase chain reaction
CCK-8: Cell counting kit-8
IgG: immunoglobulin G
GSH: Glutathione
RIP: RNA immunoprecipitation
WT: Wildtype
MUT: mutant-type
GAPDH: glyceraldehyde-3-phosphate dehydrogenase 9
ANOVA: analysis of variance
MI: myocardial infarction
